# *In silico* analyses identify gene-sets, associated with clinical outcome in ovarian cancer: role of mitotic kinases

**DOI:** 10.18632/oncotarget.8118

**Published:** 2016-03-16

**Authors:** Alberto Ocaña, Javier Pérez-Peña, Ana Alcaraz-Sanabria, Verónica Sánchez-Corrales, Cristina Nieto-Jiménez, Arnoud J. Templeton, Bostjan Seruga, Atanasio Pandiella, Eitan Amir

**Affiliations:** ^1^ Translational Research Unit, Albacete University Hospital, Albacete, Spain; ^2^ Department of Medical Oncology and Hematology, St Gallen, Switzerland; ^3^ Department of Medical Oncology, Institute of Oncology Ljubljana, Ljubljana, Slovenia; ^4^ Cancer Research Center, CSIC-University of Salamanca, Spain; ^5^ Division of Medical Oncology and Hematology, Princess Margaret Cancer Centre, University of Toronto, Toronto, Canada

**Keywords:** mitotic kinases, AURKB, protein kinases, ovarian cancer, cell cycle

## Abstract

**Introduction:**

Accurate assessment of prognosis in early stage ovarian cancer is challenging resulting in suboptimal selection of patients for adjuvant therapy. The identification of predictive markers for cytotoxic chemotherapy is therefore highly desirable. Protein kinases are important components in oncogenic transformation and those relating to cell cycle and mitosis control may allow for identification of high-risk early stage ovarian tumors.

**Methods:**

Genes with differential expression in ovarian surface epithelia (OSE) and ovarian cancer epithelial cells (CEPIs) were identified from public datasets and analyzed with dChip software. Progression-free (PFS) and overall survival (OS) associated with these genes in stage I/II and late stage ovarian cancer was explored using the Kaplan Meier Plotter online tool.

**Results:**

Of 2925 transcripts associated with modified expression in CEPIs compared to OSE, 66 genes coded for upregulated protein kinases. Expression of 9 of these genes (CDC28, CHK1, NIMA, Aurora kinase A, Aurora kinase B, BUB1, BUB1βB, CDKN2A and TTK) was associated with worse PFS (HR:3.40, log rank p<0.001). The combined analyses of CHK1, CDKN2A, AURKA, AURKB, TTK and NEK2 showed the highest magnitude of association with PFS (HR:4.62, log rank p<0.001). Expression of AURKB predicted detrimental OS in stage I/II ovarian cancer better than all other combinations

**Conclusion:**

Genes linked to cell cycle control are associated with worse outcome in early stage ovarian cancer. Incorporation of these biomarkers in clinical studies may help in the identification of patients at high risk of relapse for whom optimizing adjuvant therapeutic strategies is needed.

## INTRODUCTION

The medical treatment of ovarian cancer relies typically on untargeted therapy utilizing cytotoxic or anti-angiogenic agents [1, 2]. As such, the identification and validation of novel targets for therapeutic intervention is required.

Additionally, despite availability of validated prognostic factors such as stage, residual disease after surgery, histological type, or tumor grade, among others [1, 2], identification of patients with early stage disease who have a worse outcome is challenging and as such optimal adjuvant therapy may not be offered to such patients. Consequently, the identification of biomarkers that could help select early stage patients with worse outcome is also desirable.

Protein kinases are a family of proteins implicated in many physiological functions [3, 4]. In cancer, deregulation of their expression has been associated with oncogenic transformation [3, 4]. A particular group of these proteins is involved in the control of cell cycle and the mitotic process, allowing cells to divide [5–8]. It is hypothesized that elevated levels of kinases involved in the mitotic spindle formation may provide a more robust measure of proliferation rate and more aggressive clinical course. In addition, some of these kinases are druggable and may constitute attractive therapeutic targets.

In this article, we aimed to identify a gene signature associated with mitosis and subsequent poor prognosis in early stage ovarian cancers.

## RESULTS

### Identification of upregulated kinases by transcriptomic analyses

**Figure 1:**
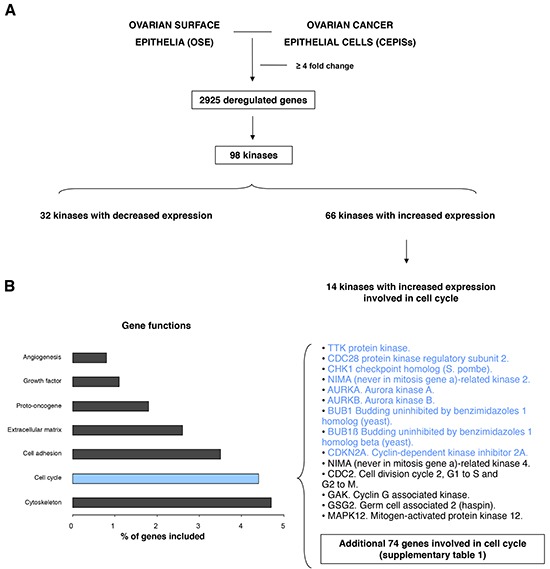
Transcriptomic expression and pathway analyses among ovarian surface epithelia (OSE) and ovarian cancer epithelial cells (CEPIs) **A.** Identification of deregulated genes with more than ≥4 fold change and selection of those that codify for kinases. **B.** Gene set enrichment analyses identify several deregulated function and list of upregulated kinases involved in cell cycle.

The initial search identified 2925 genes as deregulated. Of these, 98 coded for protein kinases with 32 being associated with reduced expression and 66 with upregulation (Figure 1A). Of these 66 upregulated kinases, 14 were associated with cell cycle functions. In parallel, using gene set enrichment analyses, we observed that cell cycle was a statistically significant gene function (Figure 1B). Among the different genes included in the cell cycle function provided by the analyses, nine were kinases upregulated in CEPIs compared with OSE. Table 1 shows a list of selected genes with the specific fold-change difference observed, including the analyses performed in our dataset and a second evaluation using oncomine. Supplementary Table 1 describes all genes included in the functional genomic analyses for cell cycle. Supplementary Table 2 describes the function of these nine selected genes.

**Table 1:** List of selected genes with the specific fold-change difference, including the evaluated dataset and validation using oncomine

Gene Name	Fold Change	P-value	Oncomine Fold Change	P-value
TTK protein kinase.	7,21	7,51E-07	15,15	2,06E-09
CKS2. CDC28 protein kinase regulatory subunit 2.	7,39	6,82E-08	5,96	3,85E-05
CHK1 checkpoint homolog (S. pombe).	4,6	4,00E-06	4,15	2,43E-07
AURKA. Aurora kinase A.	7	3,64E-08	6,5	6,53E-08
AURKB. Aurora kinase B.	5,1	1,03E-03	2,82	2,34E-05
BUB1. Budding uninhibited by benzimidazoles 1 homolog (yeast).	20,04	1,91E-07	5,27	2,15E-08
BUB1β. Budding uninhibited by benzimidazoles 1 homolog beta (yeast).	7,09	6,83E-10	8,04	2,56E-07
CDKN2A. Cyclin-dependent kinase inhibitor 2A.	45,1	2,20E-05	6,48	5,6E-14

### Association of the identified kinases with poor outcome in early stage ovarian cancer

**Figure 2:**
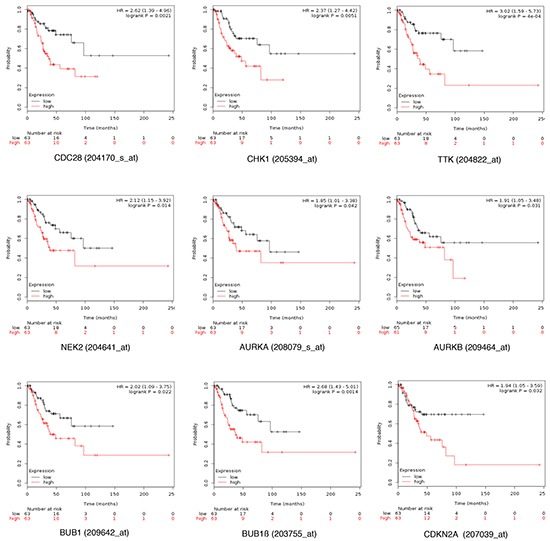
Association of CDC28, CHK1, NIMA, Aurora kinase A. Aurora kinase B. BUB1, BUB1B, CDKN2A and TTK individually with progression free survival in stage I/II ovarian cancer

**Figure 3:**
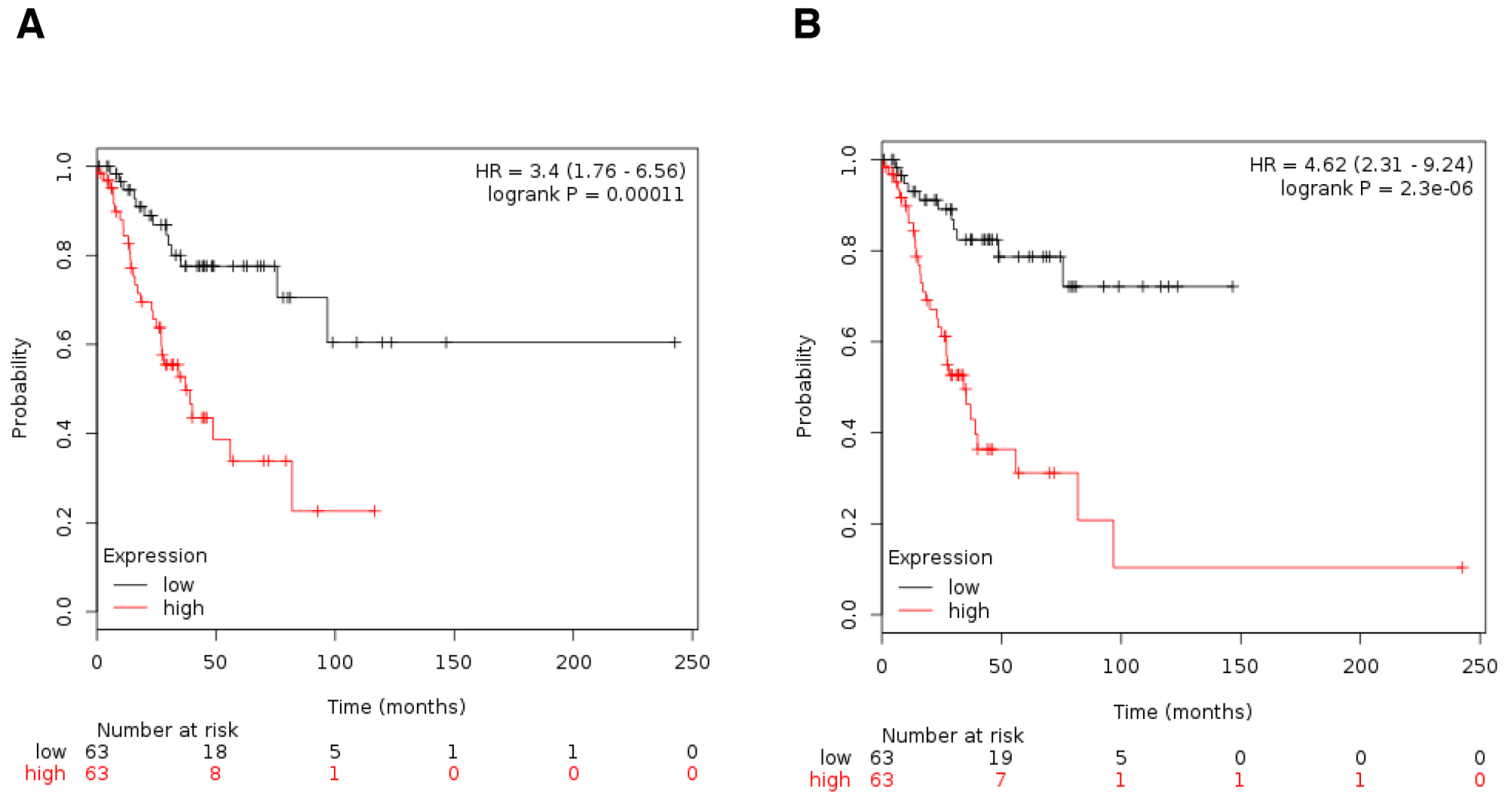
A. Association of the combined analyses of CDC28, CHK1, NIMA, Aurora kinase A, Aurora kinase B, BUB1, BUB1B, CDKN2A and TTK with progression free survival in stage I/II ovarian cancer. B. Association of the combined analyses of CHK1, CDKN2A, AURKA, AURKB, TTK and NEK2 with progression free survival in stage I/II ovarian cancer

Expression of CDC28, CHK1, NIMA, Aurora kinase A, Aurora kinase B, BUB1, BUB1β, CDKN2A and TTK were all associated with poor progression free survival (PFS) using the online tool KM plotter in stage I and II (Figure 2). In a pooled analysis, the combination of the nine genes had a numerically greater prognostic effect compared to each gene individually (Figure 3A). The combined analyses of different set of genes showed a clear association with worse outcome for some of them (Supplementary Table 3). The combined analyses of CHK1, CDKN2A, AURKA, AURKB, TTK and NEK2 showed the worse association with PFS (Figure 3B).

### Upregulation of AURKB is associated with worse OS in early stage ovarian cancer

**Figure 4:**
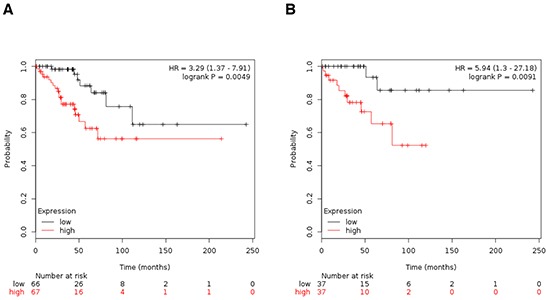
A. Association of Aurora kinase B with OS in stage I/II ovarian cancer. B. Association of Aurora kinase B with OS in stage I ovarian cancer

Analyses of AURKB showed an association with detrimental OS in stage I/II ovarian cancer to a greater extent than other combinations (Figure 4A). Similar results were obtained when patients with stage I only were analyzed (Figure 4B).

### Role of identified kinases on outcome in advanced stage ovarian cancer

**Figure 5:**
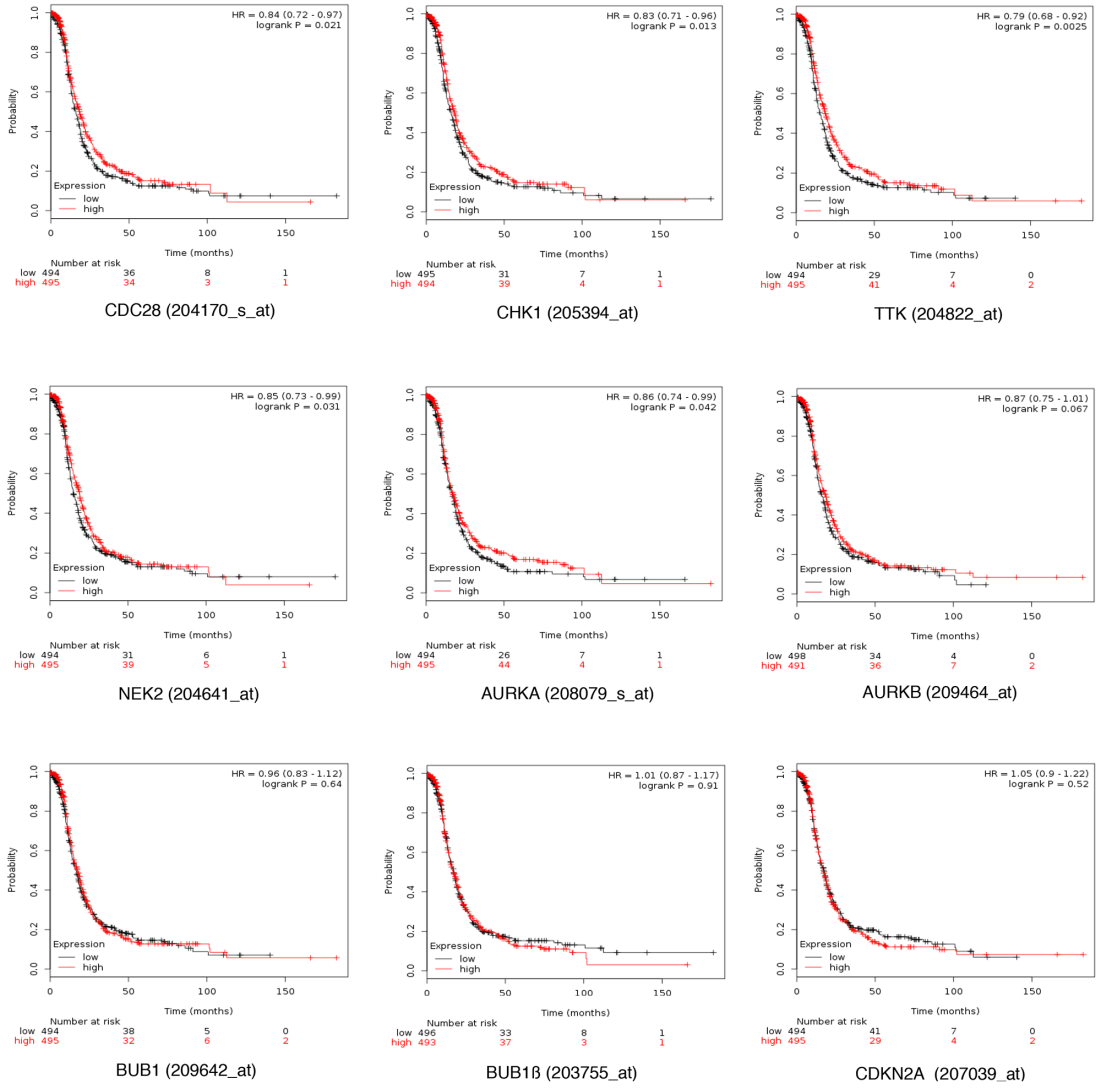
Association of CDC28, CHK1, TTK, NIMA and Aurora kinases A, B and CDKN2A individually with progression free survival in stage III and IV ovarian cancer

**Figure 6:**
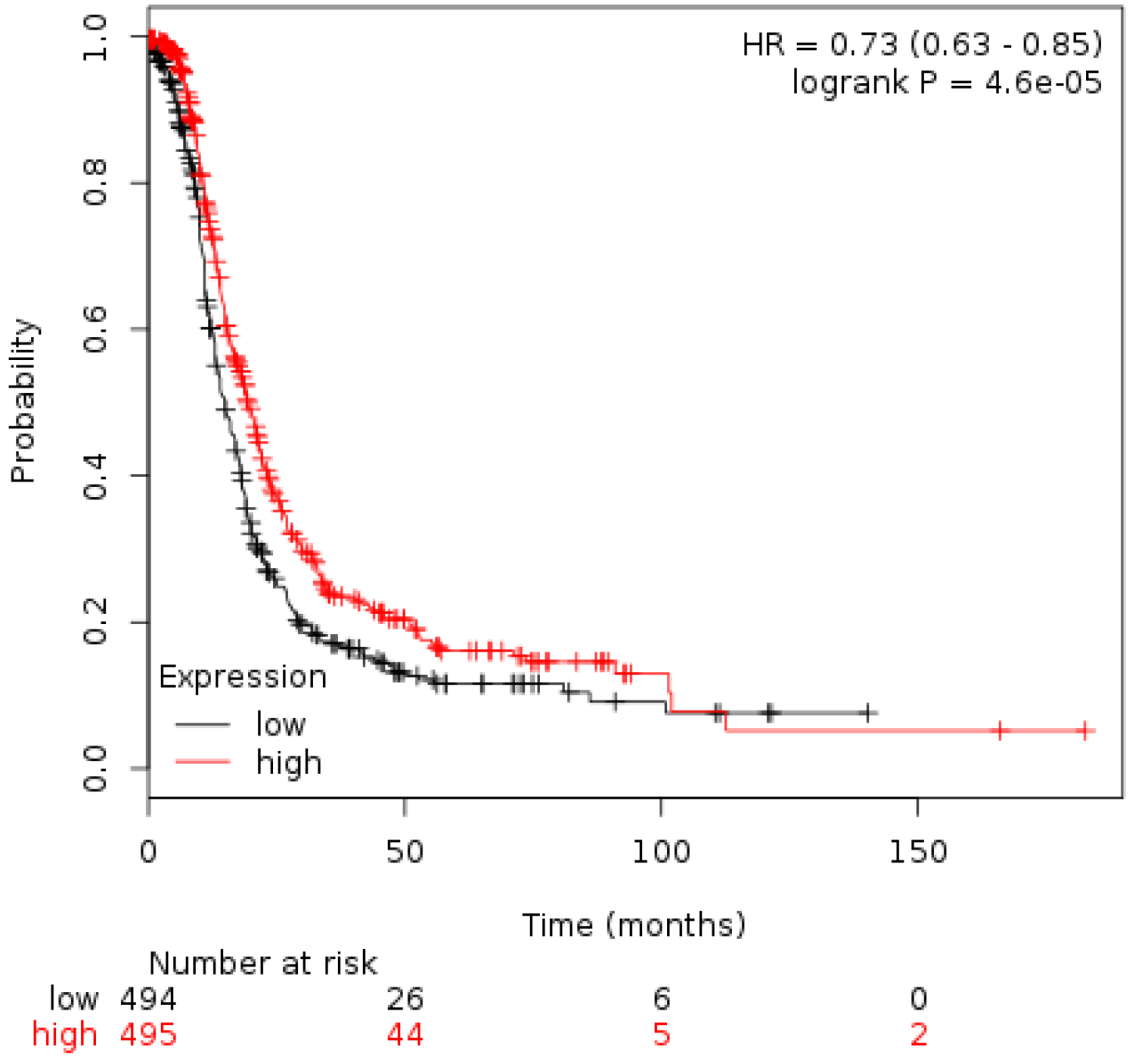
Association of the combined analyses of TTK and NIMA with progression free survival in stage III/IV ovarian cancer

In stage III and IV, expression of CDC28, CHK1, TTK, NIMA and Aurora kinase A was associated with improved PFS (Figure 5). The combined analyses of TTK and NIMA was associated with the greatest magnitude of improvement in PFS (Figure 6). No association was found for OS for any gene individually or for either combination of genes (data not shown).

### Presence of molecular alterations at a gene level

To get insights into the presence of copy number alterations or mutations in the identified genes, we used data contained at cBioportal (http://www.cbioportal.org). Table 2 describes the information identified, showing that CHK1, NIMA and AURKA are amplified in more than 3% of ovarian tumors.

**Table 2:** Information about copy number alterations or mutations in the selected genes using data contained at cBioportal

311 Ovarian Serous Cystadenocarcinoma Samples			
Gene Name	Amplification	Deletion	Mutation
TTK protein kinase.	1.3%	0.3%	0.6%
CKS2. CDC28 protein kinase regulatory subunit 2.	0.3%	-	-
CHK1 checkpoint homolog (S. pombe).	3.9%	0.6%	-
NEK2. NIMA (never in mitosis gene a)-related kinase 2.	5.1%	0.6%	-
AURKA. Aurora kinase A.	9.6%	-	-
AURKB. Aurora kinase B.	0.6%	0.6%	-
BUB1 Budding uninhibited by benzimidazoles 1 homolog (yeast).	1%	0.6%	0.3%
BUB1β Budding uninhibited by benzimidazoles 1 homolog beta (yeast).	0.6%	1.9%	0.6%
CDKN2A. Cyclin-dependent kinase inhibitor 2A.	2.3%	3.2%	-

## DISCUSSION

In the present article we describe the prognostic role of a set of genes that code for protein kinases involved in the mitotic process. A relevant finding is that some gene-sets predict detrimental PFS and OS in early stage tumors. These genes identify patients with poor prognosis and therefore tumors with such genes may be good candidates for more aggressive adjuvant therapy or considered as candidates for novel targeted therapy.

By using transcriptomic analyses, we identified upregulated kinases in ovarian tumors selecting those involved in the mitotic process. These proteins are essential for the division of cells and it is not surprising that in transformed cells their expression is biologically relevant [6]. Therefore expression of these proteins can be interpreted as a surrogate biomarker for high proliferation, showing the oncogenic addiction of these cells to the mitotic process.

When evaluating their function in clinical outcome we observed a different role in early stage tumors compared with advanced cancers. In early stage tumors, an increase expression was linked with worse PFS. Of note, the expression of AURKB was associated with worse OS in early stage diseases, including stage I alone. Patients with stage I tumors typically, do not receive adjuvant chemotherapy as a standard of care, so identification of tumors with worse prognosis could help to identify patients for more optimal adjuvant therapy. Finally, inhibition of these genes could be pharmacologically exploited. Indeed tyrosine kinase inhibitors against Aurora kinases, are currently in clinical development [9, 10].

In contrast, upregulation of five of these genes in stage III and IV ovarian cancer was linked with better PFS and no difference in OS. This finding is likely explained by the fact that in stage I and II only some patients are treated with chemotherapy, and regimens are of shorter duration, typically. Conversely, in stage III and IV the majority of patients receive longer durations of chemotherapy. As tumors with a higher proliferation rate are usually more sensitive to chemotherapy and therefore, tumors with high expression of these biomarkers are those that proliferate faster, these may also provide a predictor for benefit from chemotherapy. This hypothesis warrants testing in prospective studies of adjuvant therapy.

The poor outcome associated with expression of these kinases in early stage disease may be an indirect measure of tumor proliferation. It is unclear if the copy number alterations observed in some of the genes encoding these kinases contribute to the more aggressive phenotype. Despite this, these amplified kinases are potential candidates for target inhibition.

Our study has limitations. This is an *in silico* analysis and as such, confirmatory prospective or retrospective studies should be performed to validate these findings. Additionally, survival data are based on small number of patients and, therefore, these results should be interpreted with caution, and considered hypothesis generating. Specifically, many analyses were based on 60-70 patients and in many cases there was incomplete information about patient and tumor characteristics. As such use of multivariable analyses was not possible.

In conclusion, genes linked to cell cycle control are associated with worse outcome in early stage ovarian cancer. Incorporation of these biomarkers in clinical studies and their evaluation as potential targets is warranted, in order to customize adjuvant strategies in early stage disease or explore novel therapeutic options in advanced disease.

## MATERIALS AND METHODS

### Transcriptomic, gene-set enrichment analyses and evaluation of copy number alterations

We used a public dataset (GEO DataSet accession number: GDS3592) of mRNA level data from ovarian surface epithelia (OSE) and ovarian cancer epithelial cells (CEPIs) to identify deregulated genes. Affymetrix CEL files were downloaded and analyzed with dChip software (Dana Farber Cancer Institute, Boston, MA). Oncomine (https://www.oncomine.org/resource/login.html), was used to confirm the findings in a more extensive dataset.

Genes with different expression values in samples of OSE and CEPIs were obtained. De-regulation was defined as a ≥4 fold change between OSE and CEPIs. The list of genes was analyzed using the gene set enrichment analysis DAVID Bioinformatics Resources 6.7 in order to identify functions of these genes. We used an adjusted p-value <0.05 to select the enriched gene-sets.

The Affymetrix IDs for the nine kinases selected were: CDC28 (ID: 204170_s_at), CHK1(ID: 205394_at), NEK2 or NIMA (ID: 211080, for KM plotter: ID: 204641_at), AURKA (ID: 208079_s_at), BUB1β (ID: 203755_at), MTS1 or CDKN2A (ID: 207039_at), TTK (ID: 204822_at), AURKB (ID: 239219_at, for KM plotter: ID: 209464_at), BUB1 (ID: 209642_at).

To evaluate copy number alterations and mutations we used data contained at cBioportal (http://www.cbioportal.org).

### Outcome analyses

The Kaplan Meier (KM) Plotter Online Tool was used to analyze the relationship between the relevance of different genes' expression and patient's clinical outcome in ovarian cancer (http://www.kmplot.com), this publicly available database, which includes outcome data on 1287 ovarian cancer patients was utilized to investigate both overall survival (OS) and progression free survival (PFS) at different stages.

All analyses were performed independently by two authors (JP and VS). Discrepancies were resolved by consensus.

## SUPPLEMENTARY TABLES








