# Supplementary material for: In silico analyses identify gene-sets, associated with clinical outcome in ovarian cancer: role of mitotic kinases
Source: Oncotarget. 2016 Mar 16;7(16):22865–72. doi: 10.18632/oncotarget.8118 (PMC5008407; doi:10.18632/oncotarget.8118)
Supplement: Supplementary file 1 [file oncotarget-07-22865-s001.pdf]

SUPPLEMENTARY TABLES

Supplementary Table S1: List of genes included in the functional genomic analyses for cell cycle

See Supplementary File 1

Supplementary Table S2: Biological functions of CDC28, CHK1, NIMA, Aurora kinase A, Aurora kinase B, BUB1, BUB1B, CDKN2A and TTK

See Supplementary File 2

Supplementary Table S3: Association with PFS in early stage disease of different combinations of gene sets (p-value, long rank test)

See Supplementary File 3
