# Supplementary figures and images for: In silico analyses identify gene-sets, associated with clinical outcome in ovarian cancer: role of mitotic kinases
Source: Oncotarget. 2016 Mar 16;7(16):22865–72. doi: 10.18632/oncotarget.8118 (PMC5008407; doi:10.18632/oncotarget.8118)

## Slide 1
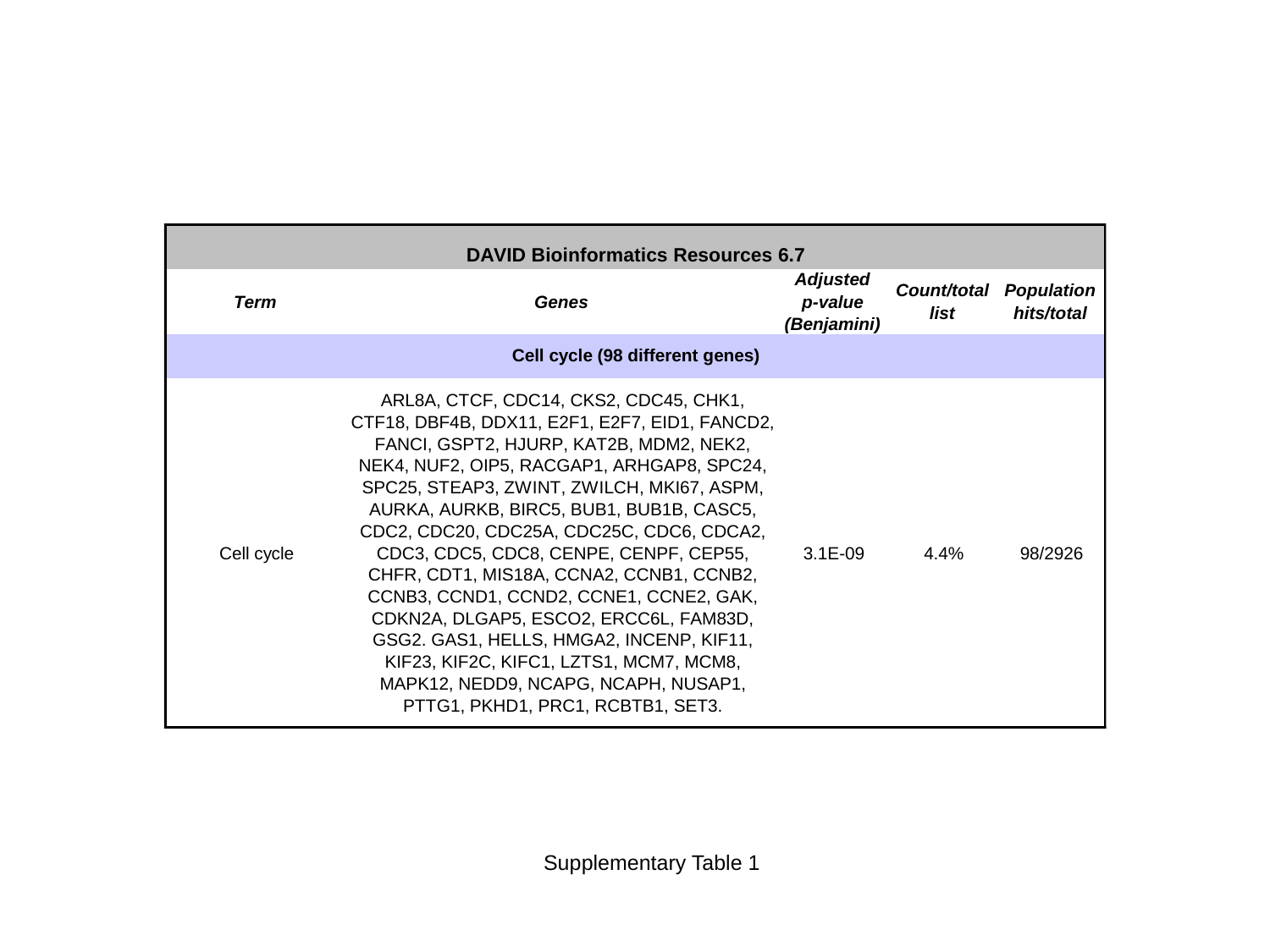

Supplementary Table 1

Supplement: Supplementary file 2 [file oncotarget-07-22865-s002.ppt]

## Slide 1
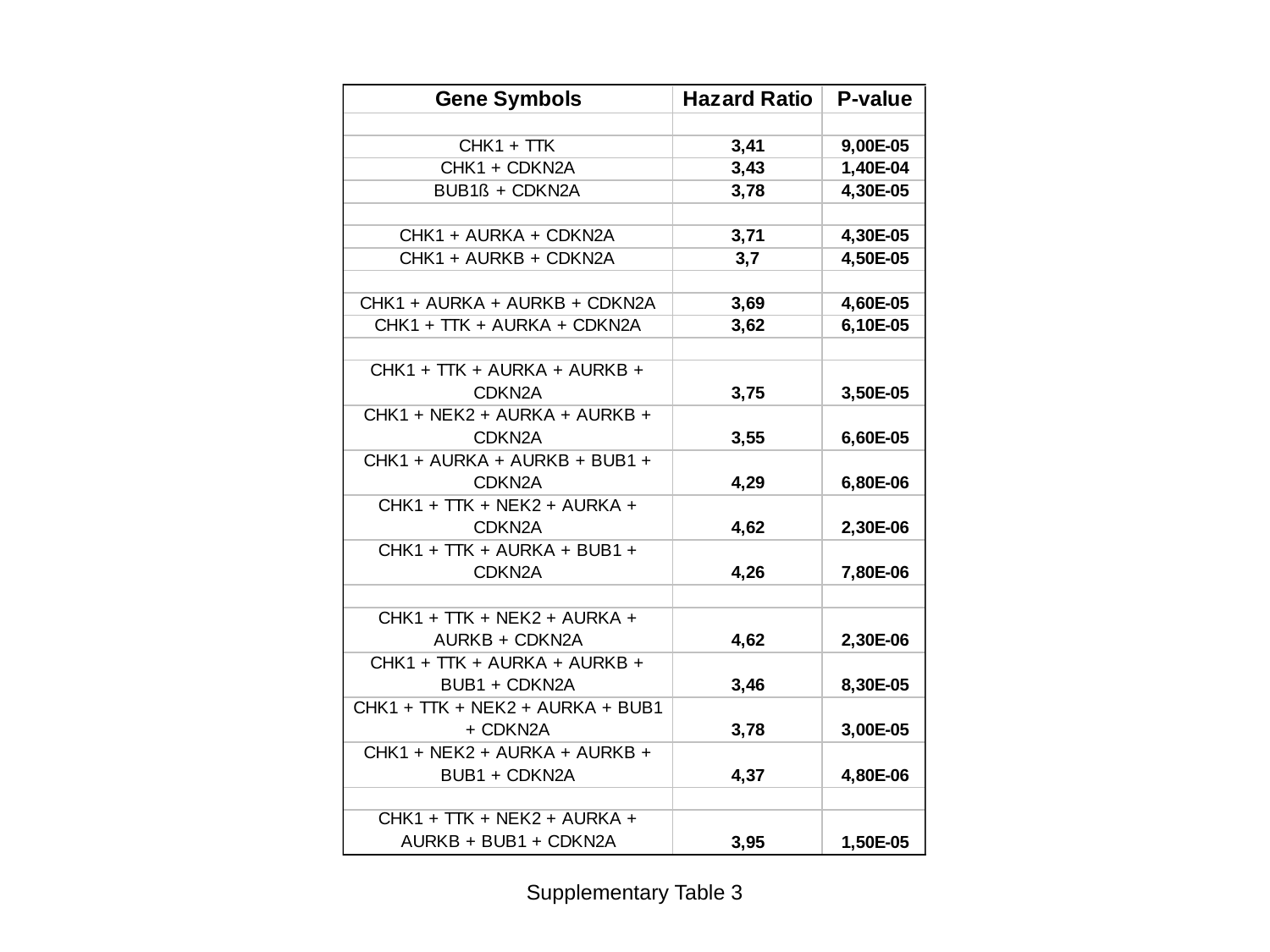

Supplementary Table 3

Supplement: Supplementary file 4 [file oncotarget-07-22865-s004.ppt]
