# Supplementary material for: In silico analyses identify gene-sets, associated with clinical outcome in ovarian cancer: role of mitotic kinases
Source: Oncotarget. 2016 Mar 16;7(16):22865–72. doi: 10.18632/oncotarget.8118 (PMC5008407; doi:10.18632/oncotarget.8118)
Supplement: Supplementary file 3 [file oncotarget-07-22865-s003.ppt]

## Slide 1
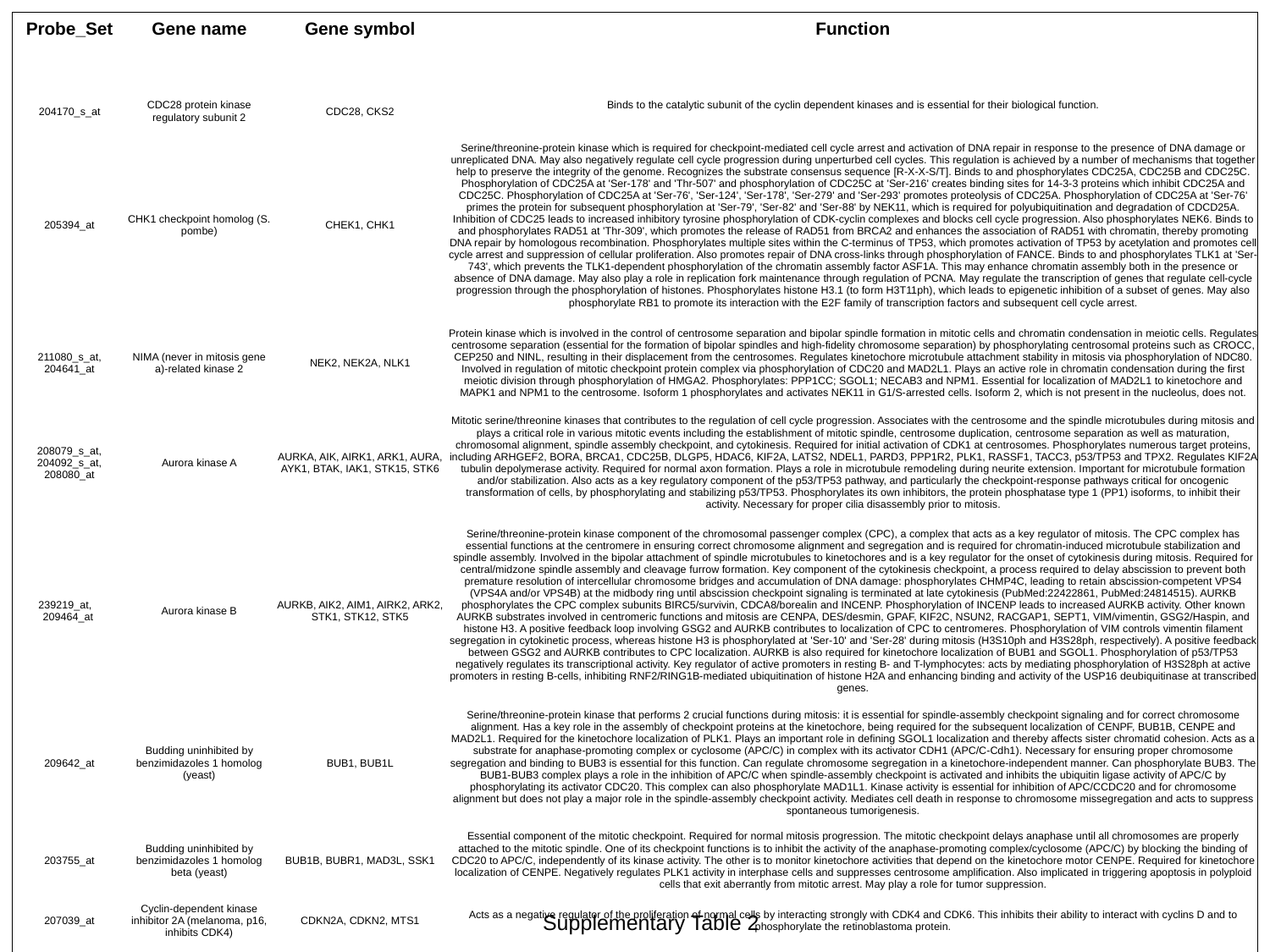

| Probe\_Set | Gene name | Gene symbol | Function |
| --- | --- | --- | --- |
| | | | |
| 204170\_s\_at | CDC28 protein kinase regulatory subunit 2 | CDC28, CKS2 | Binds to the catalytic subunit of the cyclin dependent kinases and is essential for their biological function. |
| 205394\_at | CHK1 checkpoint homolog (S. pombe) | CHEK1, CHK1 | Serine/threonine-protein kinase which is required for checkpoint-mediated cell cycle arrest and activation of DNA repair in response to the presence of DNA damage or unreplicated DNA. May also negatively regulate cell cycle progression during unperturbed cell cycles. This regulation is achieved by a number of mechanisms that together help to preserve the integrity of the genome. Recognizes the substrate consensus sequence [R-X-X-S/T]. Binds to and phosphorylates CDC25A, CDC25B and CDC25C. Phosphorylation of CDC25A at 'Ser-178' and 'Thr-507' and phosphorylation of CDC25C at 'Ser-216' creates binding sites for 14-3-3 proteins which inhibit CDC25A and CDC25C. Phosphorylation of CDC25A at 'Ser-76', 'Ser-124', 'Ser-178', 'Ser-279' and 'Ser-293' promotes proteolysis of CDC25A. Phosphorylation of CDC25A at 'Ser-76' primes the protein for subsequent phosphorylation at 'Ser-79', 'Ser-82' and 'Ser-88' by NEK11, which is required for polyubiquitination and degradation of CDCD25A. Inhibition of CDC25 leads to increased inhibitory tyrosine phosphorylation of CDK-cyclin complexes and blocks cell cycle progression. Also phosphorylates NEK6. Binds to and phosphorylates RAD51 at 'Thr-309', which promotes the release of RAD51 from BRCA2 and enhances the association of RAD51 with chromatin, thereby promoting DNA repair by homologous recombination. Phosphorylates multiple sites within the C-terminus of TP53, which promotes activation of TP53 by acetylation and promotes cell cycle arrest and suppression of cellular proliferation. Also promotes repair of DNA cross-links through phosphorylation of FANCE. Binds to and phosphorylates TLK1 at 'Ser-743', which prevents the TLK1-dependent phosphorylation of the chromatin assembly factor ASF1A. This may enhance chromatin assembly both in the presence or absence of DNA damage. May also play a role in replication fork maintenance through regulation of PCNA. May regulate the transcription of genes that regulate cell-cycle progression through the phosphorylation of histones. Phosphorylates histone H3.1 (to form H3T11ph), which leads to epigenetic inhibition of a subset of genes. May also phosphorylate RB1 to promote its interaction with the E2F family of transcription factors and subsequent cell cycle arrest. |
| 211080\_s\_at, 204641\_at | NIMA (never in mitosis gene a)-related kinase 2 | NEK2, NEK2A, NLK1 | Protein kinase which is involved in the control of centrosome separation and bipolar spindle formation in mitotic cells and chromatin condensation in meiotic cells. Regulates centrosome separation (essential for the formation of bipolar spindles and high-fidelity chromosome separation) by phosphorylating centrosomal proteins such as CROCC, CEP250 and NINL, resulting in their displacement from the centrosomes. Regulates kinetochore microtubule attachment stability in mitosis via phosphorylation of NDC80. Involved in regulation of mitotic checkpoint protein complex via phosphorylation of CDC20 and MAD2L1. Plays an active role in chromatin condensation during the first meiotic division through phosphorylation of HMGA2. Phosphorylates: PPP1CC; SGOL1; NECAB3 and NPM1. Essential for localization of MAD2L1 to kinetochore and MAPK1 and NPM1 to the centrosome. Isoform 1 phosphorylates and activates NEK11 in G1/S-arrested cells. Isoform 2, which is not present in the nucleolus, does not. |
| 208079\_s\_at, 204092\_s\_at, 208080\_at | Aurora kinase A | AURKA, AIK, AIRK1, ARK1, AURA, AYK1, BTAK, IAK1, STK15, STK6 | Mitotic serine/threonine kinases that contributes to the regulation of cell cycle progression. Associates with the centrosome and the spindle microtubules during mitosis and plays a critical role in various mitotic events including the establishment of mitotic spindle, centrosome duplication, centrosome separation as well as maturation, chromosomal alignment, spindle assembly checkpoint, and cytokinesis. Required for initial activation of CDK1 at centrosomes. Phosphorylates numerous target proteins, including ARHGEF2, BORA, BRCA1, CDC25B, DLGP5, HDAC6, KIF2A, LATS2, NDEL1, PARD3, PPP1R2, PLK1, RASSF1, TACC3, p53/TP53 and TPX2. Regulates KIF2A tubulin depolymerase activity. Required for normal axon formation. Plays a role in microtubule remodeling during neurite extension. Important for microtubule formation and/or stabilization. Also acts as a key regulatory component of the p53/TP53 pathway, and particularly the checkpoint-response pathways critical for oncogenic transformation of cells, by phosphorylating and stabilizing p53/TP53. Phosphorylates its own inhibitors, the protein phosphatase type 1 (PP1) isoforms, to inhibit their activity. Necessary for proper cilia disassembly prior to mitosis. |
| 239219\_at, 209464\_at | Aurora kinase B | AURKB, AIK2, AIM1, AIRK2, ARK2, STK1, STK12, STK5 | Serine/threonine-protein kinase component of the chromosomal passenger complex (CPC), a complex that acts as a key regulator of mitosis. The CPC complex has essential functions at the centromere in ensuring correct chromosome alignment and segregation and is required for chromatin-induced microtubule stabilization and spindle assembly. Involved in the bipolar attachment of spindle microtubules to kinetochores and is a key regulator for the onset of cytokinesis during mitosis. Required for central/midzone spindle assembly and cleavage furrow formation. Key component of the cytokinesis checkpoint, a process required to delay abscission to prevent both premature resolution of intercellular chromosome bridges and accumulation of DNA damage: phosphorylates CHMP4C, leading to retain abscission-competent VPS4 (VPS4A and/or VPS4B) at the midbody ring until abscission checkpoint signaling is terminated at late cytokinesis (PubMed:22422861, PubMed:24814515). AURKB phosphorylates the CPC complex subunits BIRC5/survivin, CDCA8/borealin and INCENP. Phosphorylation of INCENP leads to increased AURKB activity. Other known AURKB substrates involved in centromeric functions and mitosis are CENPA, DES/desmin, GPAF, KIF2C, NSUN2, RACGAP1, SEPT1, VIM/vimentin, GSG2/Haspin, and histone H3. A positive feedback loop involving GSG2 and AURKB contributes to localization of CPC to centromeres. Phosphorylation of VIM controls vimentin filament segregation in cytokinetic process, whereas histone H3 is phosphorylated at 'Ser-10' and 'Ser-28' during mitosis (H3S10ph and H3S28ph, respectively). A positive feedback between GSG2 and AURKB contributes to CPC localization. AURKB is also required for kinetochore localization of BUB1 and SGOL1. Phosphorylation of p53/TP53 negatively regulates its transcriptional activity. Key regulator of active promoters in resting B- and T-lymphocytes: acts by mediating phosphorylation of H3S28ph at active promoters in resting B-cells, inhibiting RNF2/RING1B-mediated ubiquitination of histone H2A and enhancing binding and activity of the USP16 deubiquitinase at transcribed genes. |
| 209642\_at | Budding uninhibited by benzimidazoles 1 homolog (yeast) | BUB1, BUB1L | Serine/threonine-protein kinase that performs 2 crucial functions during mitosis: it is essential for spindle-assembly checkpoint signaling and for correct chromosome alignment. Has a key role in the assembly of checkpoint proteins at the kinetochore, being required for the subsequent localization of CENPF, BUB1B, CENPE and MAD2L1. Required for the kinetochore localization of PLK1. Plays an important role in defining SGOL1 localization and thereby affects sister chromatid cohesion. Acts as a substrate for anaphase-promoting complex or cyclosome (APC/C) in complex with its activator CDH1 (APC/C-Cdh1). Necessary for ensuring proper chromosome segregation and binding to BUB3 is essential for this function. Can regulate chromosome segregation in a kinetochore-independent manner. Can phosphorylate BUB3. The BUB1-BUB3 complex plays a role in the inhibition of APC/C when spindle-assembly checkpoint is activated and inhibits the ubiquitin ligase activity of APC/C by phosphorylating its activator CDC20. This complex can also phosphorylate MAD1L1. Kinase activity is essential for inhibition of APC/CCDC20 and for chromosome alignment but does not play a major role in the spindle-assembly checkpoint activity. Mediates cell death in response to chromosome missegregation and acts to suppress spontaneous tumorigenesis. |
| 203755\_at | Budding uninhibited by benzimidazoles 1 homolog beta (yeast) | BUB1B, BUBR1, MAD3L, SSK1 | Essential component of the mitotic checkpoint. Required for normal mitosis progression. The mitotic checkpoint delays anaphase until all chromosomes are properly attached to the mitotic spindle. One of its checkpoint functions is to inhibit the activity of the anaphase-promoting complex/cyclosome (APC/C) by blocking the binding of CDC20 to APC/C, independently of its kinase activity. The other is to monitor kinetochore activities that depend on the kinetochore motor CENPE. Required for kinetochore localization of CENPE. Negatively regulates PLK1 activity in interphase cells and suppresses centrosome amplification. Also implicated in triggering apoptosis in polyploid cells that exit aberrantly from mitotic arrest. May play a role for tumor suppression. |
| 207039\_at | Cyclin-dependent kinase inhibitor 2A (melanoma, p16, inhibits CDK4) | CDKN2A, CDKN2, MTS1 | Acts as a negative regulator of the proliferation of normal cells by interacting strongly with CDK4 and CDK6. This inhibits their ability to interact with cyclins D and to phosphorylate the retinoblastoma protein. |
| 204822\_at | TTK | TTK, MPS1, MPS1L1 | Phosphorylates proteins on serine, threonine, and tyrosine. Probably associated with cell proliferation. Essential for chromosome alignment by enhancing AURKB activity (via direct CDCA8 phosphorylation) at the centromere, and for the mitotic checkpoint. |
Supplementary Table 2
